# The Importance of Digital Rectal Examination in Identifying Early-Onset Rectal Malignancies

**DOI:** 10.7759/cureus.59458

**Published:** 2024-05-01

**Authors:** Anshul Ramanathan, Benjamin Smith

**Affiliations:** 1 Medicine, Edward Via College of Osteopathic Medicine, Blacksburg, USA; 2 Surgery, LewisGale Medical Center, Blacksburg, USA

**Keywords:** colorectal cancer, post-grad medical education, screening colonoscopy, internal hemorrhoids, per-rectal bleeding, diagnostic colonoscopy, rectal exam

## Abstract

Colorectal cancer (CRC) is the second most diagnosed cancer and the second leading cause of cancer-related deaths in the United States. Rectal cancers, specifically, are the second most common cancer of the large intestine. Although once perceived as a disease of the elderly, the incidence of early-onset CRC (EO-CRC), classified as occurring in individuals less than 50 years old, has been paradoxically increasing. Although the incidence of rectal cancers has increased, the digital rectal exam (DRE) continues to be an underutilized physical exam maneuver when a patient presents with red-flag symptoms. Here, we present a case of a 38-year-old male from West Virginia who was referred to general surgery for complaints of rectal bleeding attributed to internal hemorrhoids. After undergoing a colonoscopy, the patient was found to have a rectal mass consistent with adenocarcinoma. We describe the importance of identifying red-flag signs to keep colorectal malignancy in the differential diagnosis in a young patient and highlight the importance of performing rectal exams to identify rectal cancers early to expedite treatment.

## Introduction

The pathogenesis of colorectal cancers (CRCs) is best described by the adenoma-carcinoma sequence, which details the progressive accumulation of mutations in oncogenes [1]. A mutation in the adenomatous polyposis coli (APC) tumor-suppressor gene, which leads to a loss of cellular adhesion and an increase in cellular proliferation, leads to the formation of polyps. This is followed by a mutation in the KRAS proto-oncogene, leading to the loss of cell-cycle regulatory proteins and a further increase in the size of adenomas [1]. Subsequent mutations in the TP53 tumor suppressor, which controls the G1 to S phase transitioning of the cell cycle, and DCC tumor suppressor, which normally induces apoptosis, further contribute to the malignant transformation of adenomas into adenocarcinomas [1]. Low-risk polyps are characterized as pedunculated, sessile, and less than 10 mm in diameter. High-risk polyps may have ulcerations and are greater than 10 mm in diameter.

The incidence of rectal cancer in people less than 50 has increased by 2.6% per year from 1984 to 2020, making screening for these malignancies even more critical in the young population [2]. In an average-risk patient, screening colonoscopies for rectal cancer should start by age 45 and be repeated every 10 years. Rectal cancers can cause symptoms, such as hematochezia, abdominal pain, and changes in stool caliber, due to the mass effect. A patient with any of these red-flag symptoms should undergo a colonoscopy to rule out malignancy. Currently, digital rectal exams (DREs) are an often-overlooked part of the physical exam and are no longer performed in every patient [3]. Many general practitioners do not routinely perform DREs either due to a lack of comfort or lack of time. A retrospective study noted that out of 262 patients, 121 (46.2%) patients did not recall having a DRE done when they presented with blood in the stool [4]. Given that many endoscopists triage based on comments in referrals, descriptions of a “palpable mass” could prompt an expedited workup by a specialist due to suspicion of rectal cancer [4]. In the same retrospective study, having a DRE performed by the general practitioner tended to lead to shorter wait times for being seen by a specialist [4].

Patients with rectal cancers should undergo CT of the chest, abdomen, and pelvis to check for metastatic disease. An MRI is also done to stage the cancer. Because of the portal venous system, the upper 2/3 of the rectum drains into the liver via the superior rectal vein. Therefore, the liver is one of the main sites for metastasis [5]. Venous drainage for the lower 1/3 of the rectum drains via the inferior rectal vein into the systemic circulation, thereby leading to metastasis to the lungs [6].

## Case presentation

A 38-year-old Caucasian male with no past medical or surgical history presented to the clinic with complaints of what he believed to be hemorrhoidal issues. In early June, the patient noted some constipation and “streaks of blood” in his stool. He visited his primary care physician (PCP) about a week later. The patient noted that although a DRE was attempted, it was not performed successfully due to both provider and patient discomfort. Since the PCP was only able to enter about ½ an inch from the anal verge, she attributed the pain and rectal bleeding to internal hemorrhoids and prescribed a benzocaine suppository. The patient reported continued pain and bleeding despite the benzocaine. On October 30, the patient experienced significant bleeding with multiple blood clots. He was evaluated in the emergency room, where, despite the symptoms, a DRE was not performed; a CT scan was conducted and returned negative results. He had never undergone a colonoscopy and denied any family history of CRC.

**Figure 1:**
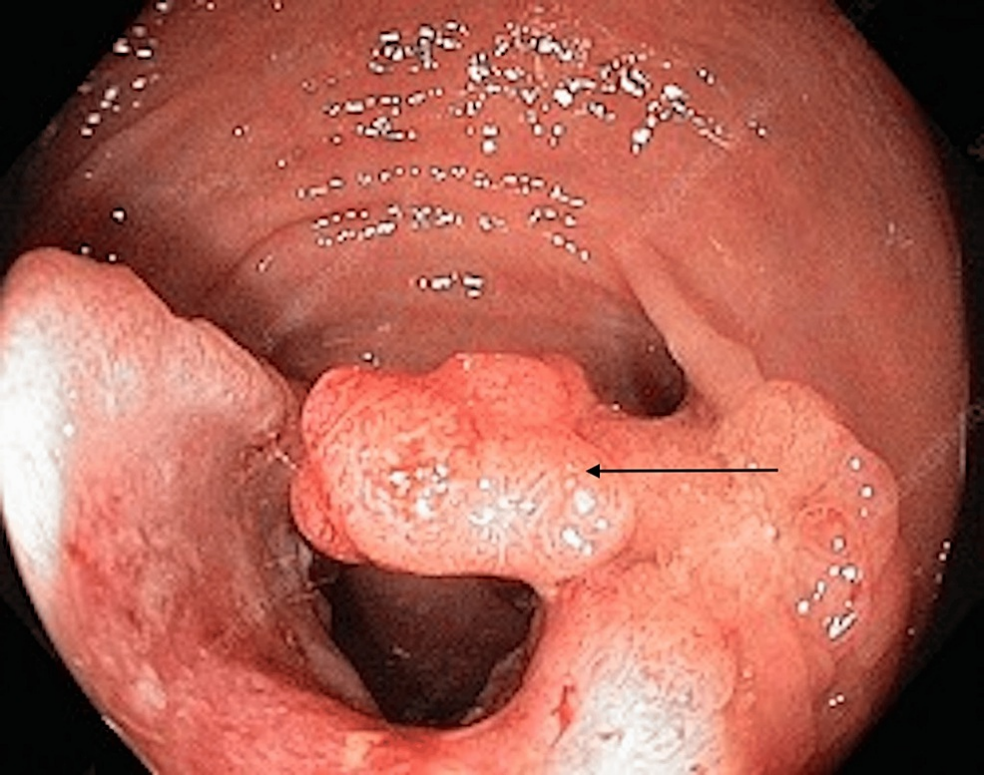
This image depicts approximately what the mass looked like under colonoscopy, around 3 cm from the anal verge. This image was obtained from a public domain.

Following the recent bleeding episode, the patient set up an appointment with a general surgeon in November, and a diagnostic colonoscopy was recommended. The procedure revealed a non-circumferential, non-obstructive, fungating, medium-sized mass located 2-3 cm from the anal verge (Figure 1).

The mass was biopsied using cold forceps, and the pathology report confirmed adenocarcinoma of the rectum. The patient was referred to hematology/oncology and scheduled for an MRI to determine staging and a PET-CT scan to check for areas of increased metabolic activity from tumor metastases.

**Figure 2:**
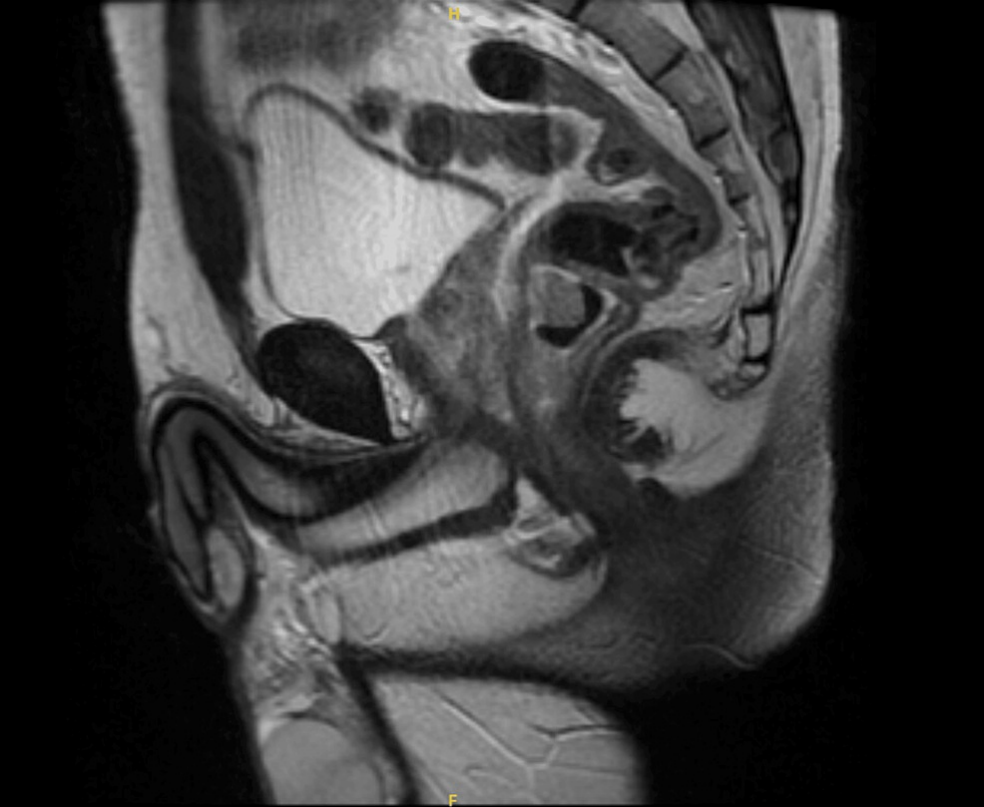
Normal MRI showing no involvement of perirectal fascia.

**Figure 3:**
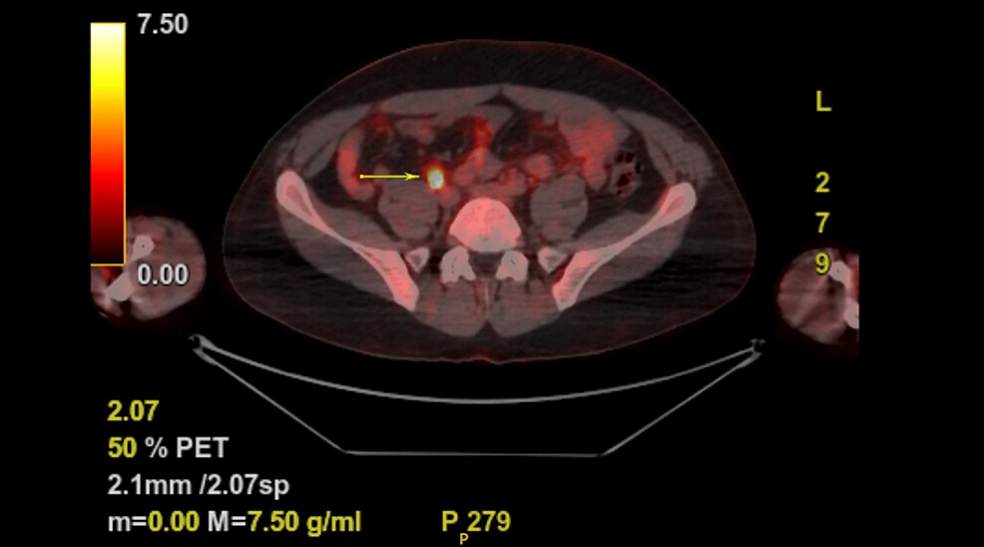
Fused axial PET/CT corresponding to a lesion in the rectum (arrow), which proved to be the site of the 2-3 cm rectal mass.

The MRI revealed no appreciable extension of the mass into the perirectal fascia (Figure 2). The PET-CT scan showed no abnormal activity in the head, neck, chest, abdomen, pelvis, or skeleton. There was a rectal lesion measuring 19.5 SUV (standardized uptake value) consistent with a history of rectal carcinoma (Figure 3). 

## Discussion

While the incidence and mortality of CRC have been decreasing in adults over 50, Surveillance Epidemiology and End Results (SEER) data from 1984 to 2020 showed a 2.6% annual increase in CRC incidence in adults under 50 [2]. This population is not routinely screened for rectal cancer, so this diagnosis may frequently be missed [7]. Even more concerning is the fact that this younger subset of the population tends to present with more advanced stages of CRC [7].

The DRE is an underutilized tool by primary care physicians and can contribute to delays in seeking definitive, life-saving care in rectal cancer patients [4]. Although most medical students are taught how to properly perform a DRE in their pre-clinical years, there is limited teaching on DRE during the clinical years and in residency [8]. A study in Australia notes that low levels of confidence and comfort in performing rectal exams were the main reasons for underutilization, particularly by doctors-in-training [9]. These doctors reported that formal training in DRE during medical school and adequate support from senior colleagues were key contributors to their comfort level in performing rectal exams [9]. Furthermore, being comfortable with performing DREs was associated with greater confidence in diagnosing benign or malignant pathology [9]. Medical school preceptors and residency program directors should ensure greater hands-on training in successfully performing a DRE so that doctors-in-training can get more comfortable in this skill and use this skill more often. If DREs become a more common practice in the physical examination of patients presenting with red flag symptoms, such as hematochezia, changes in stool caliber, or iron-deficiency anemia in men, then physicians will be better equipped to identify rectal malignancies in their early stages.

## Conclusions

CRC can be asymptomatic early on, but it commonly presents with red-flag symptoms, such as changes in bowel habits, hematochezia, anemia, and weight loss. Given the high incidence of rectal cancers and the glaring symptoms they may present, performing a rectal exam is paramount to ruling out malignancy. A rectal exam conducted when the patient first presented to the physician in June 2023 could have potentially diagnosed the cancer at least two to three months earlier. As important as rectal exams are, many providers endorse a lack of comfort with performing rectal exams due to inadequate training during their clinical and post-graduate years. Further steps need to be taken to ensure that there is structured formal training for DREs during the clinical years and post-graduate years of medical training.
